# Tremor and Dysmetria in Multiple Sclerosis: A Neurophysiological Study

**DOI:** 10.5334/tohm.598

**Published:** 2021-07-26

**Authors:** S. H. Alusi, A. Macerollo, Colum D. MacKinnon, John C. Rothwell, Peter G. Bain

**Affiliations:** 1 Department of Neurology, The Walton Centre NHS Foundation Trust, Liverpool, UK; 2 Faculty of Health and Life Sciences, University of Liverpool, UK; 3 Department of Neurology, University of Minnesota, Minneapolis, MN, USA; 4 UCL Queen Square Institute of Neurology, London, UK; 5 Imperial Centre for Restorative Neuroscience, Imperial College London, London, UK

**Keywords:** Tremor, Multiple Sclerosis, Neurophysiology

## Abstract

**Objective:**

The mechanisms contributing to the pathogenesis of tremor and/or dysmetria in multiple sclerosis (MS) are poorly understood. Abnormal oscillations within the olivo-cerebello-thalamo-cortical networks are believed to play an important part in tremor aetiology, but could also contribute to intention dysmetria due to disruptions in motor timing. Conversely, delayed central motor conduction times are a common feature of ataxias, but could also contribute to the expression of dysmetria in MS. This study examined the roles of central conduction delays in the manifestation of tremor and/or dysmetria in MS.

**Methods:**

Twenty-three individuals with MS participated: 8 with no movement disorder, 6 with tremor, 4 with pure dysmetria and 5 with both tremor and dysmetria. Median nerve somatosensory evoked potentials (SEPs), transcranial magnetic stimulation (TMS) over the motor cortex and cervical spine, stretch reflexes were used assess sensory and motor conduction times.

**Results:**

Central, but not peripheral, sensory conductions time were significantly delayed in participants with dysmetria, regardless of the presence of tremor. Similarly, the TMS evoked muscles responses and the long-latency component of stretch reflexes were significantly delayed in those with dysmetria, but not pure tremor.

**Conclusion:**

Dysmetria in MS is associated with delays in central conduction of sensory or motor pathways, or both, likely leading to disruption of muscle activation timing and terminal oscillations that contribute to dysmetria.

**Significance:**

The presence of dysmetria in MS is associated with decreased conduction velocities in central sensory and/or motor pathways likely reflects greater demyelination of these axons compared to those with no movement disorder or pure tremor.

## Introduction

The neurophysiology of tremor and dysmetria in multiple sclerosis (MS) is poorly understood. People with MS can present with tremor, dysmetria, or both. Lesions in cerebello-thalamocortical pathways and/or central sensory and motor pathways could contribute to the pathogenesis of either tremor or dysmetria. Pathological oscillations within the olivo-cerebello-thalamo-cortical network are believed to play an important role on tremor aetiology in other tremor pathologies [1,2,3]. For example, the cerebellum is considered to play a central role in the pathogenesis of tremor in people with essential tremor (ET) [4]. Yet, the majority of these individuals do not show signs of dysmetria, suggesting that central cerebellar circuitry may not contribute to dysmetria. In contrast, dysmetria in people with ataxias is associated with delays in central sensory conduction times (CSCTs), as assessed using somatosensory evoked potentials (SEPs). Similarly, central motor conductions times (CMCTs), assessed using transcranial magnetic stimulation (TMS), are abnormally prolonged in some forms of ataxia (e.g. Friedrich’s ataxia, spinocerebellar ataxia type 1) but no other forms [4]. However, dysfunction and delays in cerebello-thalamocortical pathways can also contribute to dysmetria [5]. For example, lesions of the deep cerebellar nuclei can result in abnormalities in muscle activation resulting in terminal oscillations [6,7,8,9]. To date, no study has examined the role of central conduction delays in the pathogenesis of tremor or dysmetria in people with MS.

Sensorimotor conduction pathways have been extensively tested in people with MS. Abnormally delayed SEPs occur in 59% of patients with MS [10] and up to 42% of these individuals have no sensory symptoms [10,11]. Similarly, descending motor conduction delays are common in MS. The sensitivity of magnetic stimulation for detecting central nervous system lesions in MS patients is relatively high ranging from 79% [12] to 88.6% [13]. Cowan et al. demonstrated that unilateral or bilateral motor conduction delays can be present in MS patients without significant evidence of motor neuron deficit [14] and the magnitude of the conduction delay did not relate to the severity of clinical deficits. However, the number of limbs with abnormal CMCT has been shown to correlate with the disease’s severity [13]. Previous studies reported prolonged CMCT’s in 28% of limbs with normal examination and in 58- 69% of limbs with sensory impairment or cerebellar signs [13,15]. Abnormal CMCT’s were found in 85% of limbs with positive pyramidal signs and in 100% of the limbs in patients with severe paresis [13]. Delays in sensory or motor conduction could result in dyscoordination of muscle activity timing during voluntary movements leading to dysmetria and end-point oscillations that resemble intention tremor.

Peripheral and central conduction times can also be assessed using stretch reflexes evoked by electrical stimulation of a peripheral nerve or imposed rapid stretch of a muscle. The short latency component (M1) of the stretch reflex response is generated by a spinal reflex pathway, whereas longer latency responses (M2 and M3) are mediated, in part, by a transcortical pathway via the motor cortex [16]. Friedman et al. reported that in patients with cerebellar disorders leading to dysmetria and ataxia, the magnitude of the long latency reflexes can be exaggerated, but the latency of M1 and M2/M3 responses were normal unless there was additional brain stem involvement when the latencies were slightly prolonged [17]. These findings demonstrate that dysmetria can arise of delays cerebellar-thalamocortical circuity in the absence of sensory or motor conduction delays.

The aim of this study was to investigate the state of the sensory and motor pathways in MS patients with tremor and dysmetria by studying the CSCTs, CMCTs and the afferent and efferent loop time obtained from stretch reflexes data, in the hope of elucidating the pathophysiology underlying these movement disorders. We hypothesized that dysmetria in MS would be associated with significantly increased central conductions times (CSCT, CMCT and M2 onsets) compared with the MS control and pure tremor groups.

## Material and Methods

Twenty-three (14 male, 9 female) MS patients were studied (***Table 1***). Their average age was 45 years (range 22–66) and average disease duration was 14 years (range 4–31). Patients were classified according to the predominant movement disorder in the studied arm: eight had no movement disorders in the arms (MS controls, MS-C n = 8), six had pure tremor (MS-T, n = 6), four had pure dysmetria (MS-D, n = 4), and five had both tremor and dysmetria (MS-M, n = 5). While the subgroup sample sizes were relatively small, the cohort allowed post-hoc comparison of the outcome variables between patients with dysmetria (MSwD: MS-D + MS-M; n = 9) and without dysmetria (MSwoD: MS-C + MS-T; n = 14). All the patients had normal power in the studied arms except for two individuals who had mild weakness (MRC power grade = 4/5). Fifteen patients had evidence of sensory impairment. The tremor severity was assessed using the (0–10) Bain and Findley Tremor Severity Scale [30] which has been validated for the assessment of tremor in MS [18]. The average score of the postural tremor severity was taken between tremor on posture 1 with the arms extended and posture 2 with the arms flexed at the elbow. Dysmetria was assessed using a 0–4 scale [18]. There was no clinical evidence of peripheral neuropathy in any of the patients. Three patients were on propranolol for the treatment of their tremor.

**Table 1:** Patients’ clinical data.


PATIENT	AGE	SEX	GROUP	HANDEDNESS	SITE TESTED	TREMOR	DYSMETRIA	POWER	SENSATION

1	51	F	C	L	R	0	0	N	I

2	59	F	C	R	R	0	0	N	N

3	61	F	C	R	R	0	0	N	N

4	40	M	C	L	L	0.5	0	N	N

5	42	F	C	L	L	0.5	0	N	N

6	53	M	C	R	R	0.5	0	N	N

7	66	M	C	R	R	0	0.5	N	N

8	45	M	T	L	L	1.5	0	N	N

9	35	F	T	R	L	4.5	1	N	N

10	36	F	T	R	R	3	1	N	I

11	55	F	T	R	L	1.5	0	N	I

12	39	F	T	R	R	0	1	N	I

13	36	M	T	L	R	3.5	1	N	I

14	31	M	D	R	R	0	1	N	I

15	31	F	D	R	L	0	2	4/5	I

16	33	M	D	R	L	0	2	N	I

17	36	M	D	R	R	0	2	N	I

18	51	M	M	R	R	5	2	4/5	I

19	56	M	M	L	L	7	1	N	I

20	37	M	M	R	L	2.5	2	N	N

21	52	M	M	R	R	1.5	1	N	I

22	22	M	M	R	R	2	1	N	N

23	58	M	C	R	R	0.5	0	N	I


C = MS control group; T = MS tremor group; D = MS dysmetria group; M = MS mixed group; N = normal; I = impaired.

### Somatosensory evoked potentials

Somatosensory evoked potentials (SEPs) were recorded from C3’ and C4’ electrodes placed over the contralateral scalp, 2 cm posterior to C3 and C4 (international 10/20 system) with reference to the ear lobes after electrical stimulation of the median nerve at the wrist using either a 200 or 500 microsecond pulse width. The stimulus intensity was set to be just above the motor threshold. Recordings were also taken from Erb’s point and the neck over the level of the fourth cervical vertebra. In each patient, a series of at least 400 trials was averaged. SEPs were amplified and filtered (time constant = 3 s, low pass filter = 3 kHz, gain of 20 microvolts per volt) and sampled at 4000 Hz. The latencies to the first negative deflection at Erb’s point, cervical cord and cortex were measured. Central sensory conduction time was calculated by subtracting the cervical spine latencies from the cerebral cortex latencies N19-N13 [10]. Central sensory conduction time was considered abnormal if it exceeded 7.1 ms [19].

### Magnetic stimulation

Magnetic stimulation of the motor cortex and cervical spine were performed using Novamatrix Magstim 200 (Magstim Co. Ltd., Whitland Dyfed UK) with a round coil with an external diameter of 12 cm. The coil was placed over the vertex in a position that stimulation activated the first dorsal interosseous (FDI) muscle of the target limb. Bipolar electromyographic (EMG) activity was amplified and captured at 4000 Hz (Gould Electronics ES2000). The threshold stimulation intensity for producing motor evoked potential (MEP) in FDI was identified. The stimulation intensity was then gradually increased from 10% below threshold in 4–5 increments of 5% of the stimulator output. Ten trials of TMS were applied at each stimulus intensity. To stimulate the cervical roots, the coil was applied over C7. Stimulation intensities were lower than those required for the cortex and were also increased by 4 increments consisting of 5% of the stimulator output. Patients were tested both at rest and with the thumb muscles minimally activated. The CMCTs were calculated as the difference in the latencies obtained from cortical stimulation and cervical cord stimulation. Normal values were obtained from the study by Mayr et al. based data from 86 healthy controls [13]. A CMCT value of more than 9.48 with lightly activated arm muscles was considered abnormal.

### Stretch reflexes

Rapidly imposed wrist extensor displacements, produced by a servomotor-driven manipulandum, were used to evoke stretch reflexes in the wrist flexors (FCR). The semi-pronated forearm rested on a platform, with the fingers encased in a rigid splint, attached to a motor (Printed Motors Type G12M) and the wrist aligned with the motor shaft. The forearm was secured to allow movements at the wrist joint only. The subjects were asked to hold their forearm in a constant position with reference to an oscilloscope display against a standing torque of 0.38 Nm. Stretches were evoked in response to three applied torque magnitude conditions: 0.76, 1.52 or 2.28 Nm. The order of the torque conditions was randomized across subjects. For each torque condition, stretches were applied every 5 seconds in batches of 32 trials. The order of the three magnitude triggers was randomized. Bipolar surface EMG recordings (8mm silver/silver-chloride electrodes) were obtained from the biceps (BIC), flexor carpi radials (FCR) and extensor digitorum (EDC) muscles. The joint angular position, the joint velocity and rectified EMG were recorded. The response signals were amplified and filtered (time constant = 3 ms, low-pass filter = 1kHz) and sampled at 2 kHz per channel. The onset and latencies of the stretch reflexes were measured by visual inspection of the averaged EMG signals. The magnitude of the M1 and M2 responses were measured by calculating the area under the rectified EMG signal for 20 ms after the onset of the response. M1 and M2 onset latencies and magnitudes were determined for each stretch load.

### Statistical analysis

A univariate analysis of variance (ANOVA) was used to test for significant differences between groups in sensory conduction times and the onset and magnitudes of the M1 and M2 stretch reflex responses. Since the MEP onset data did not meet criteria for a normal distribution, the data were log-transformed. The absence of a detectable MEP response from some muscle groups of some patients meant that there was a number of missing values which made the data unsuitable for a standard analysis of variance calculation. A Residual Maximum Likelihood (REML) procedure was used as the method of analysis [20]. For the stretch reflex data, a repeated measures general linear model with between-group factors of group and repeated factor of torque pre-load (low, medium, high). Post-hoc analysis of group or interaction-effects were conducted using Tukey’s Honestly Significant Difference test.

## Results

### SEPs

**Figure 1:**
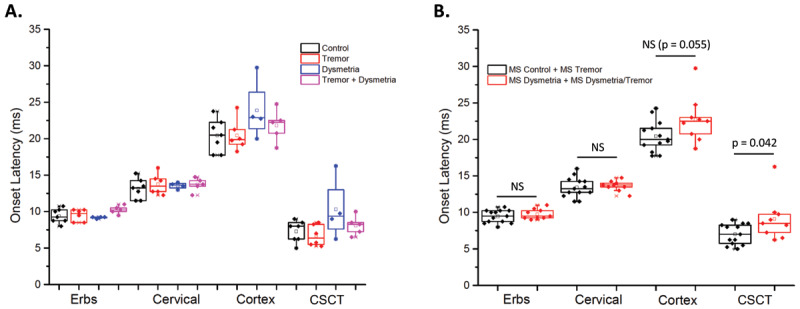
**A.** Onset latency of the sensory evoked potentials recorded at Erbs point, cervical spinal cord and cortex and the central sensory conduction times (CSCT) for each MS subgroup. The boxes show the 75^th^ and 25^th^ percentiles and median, the open box shows the mean value and data points with an X show the maximum and minimum values. **B.** Same data as in A, but the groups have been combined into those with dysmetria (red) and without dysmetria (black). NS = no significant difference between groups.

The latencies to Erb’s point, cervical spine and cerebral cortex are summarized in ***Figure 1***. Latencies to Erb’s point were within normal range in all patients, confirming the absence of peripheral sensory nerve pathology. The average CSCTs for the four groups were: (MS controls, MS tremor, MS dysmetria and MS tremor + dysmetria) were MS-C = 7.29 ± 1.46 ms, MS-T = 6.7 ± 1.43 ms. MS-D = 10.31 ± 4.23 ms, MS-M = 8.1 ± 1.32 ms (***Table 2***). ANOVA that compared across all four MS groups showed no significant effects of group (p > 0.095). When the combined groups of participants with dysmetria and without dysmetria were compared, there was a significant difference between groups in CSCT (F = 4.730, p = 0.042) and difference in onset time of the cortical evoked neared significance threshold (F = 4.135, p = 0.055). On average, the CSCT times were 2.1 ms (28%) longer in the group with dysmetria compared with the group without dysmetria.

**Table 2:** SEPs latencies for the four groups.


PATIENT	GROUP	ERB’S POINT (LATENCIES, ms)	CERVICAL SPINE (LATENCIES, ms)	CEREBRAL CORTEX (LATENCIES, ms)	CENTRAL CONDUCTION TIMES (ms)

1	C	8.0	11.5	17.75	6.25

2	C	9.25	12.75	17.75	5

3	C	10.25	13.25	19.5	6.25

4	C	8.75	14.25	22.25	8

5	C	8.75	11.5	20.5	9

6	C	10.0	15.25	23.75	8.5

7	C	10.75	13.5	21.5	8

8	T	10.0	14.25	20.0	5.75

9	T	9.5	12.75	21.25	8.5

10	T	8.5	12.75	18.25	5.5

11	T	10.25	14.5	19.75	5.25

12	T	8.5	12.25	19.25	7

13	T	10.25	16.0	24.25	8.25

14	D	9.25	13.0	22.75	9.75

15	D	9.0	14.0	23.0	9

16	D	9.25	13.75	20.0	6.25

17	D	9.25	13.5	29.75	16.25

18	M	10.25	14.25	22.5	8.25

19	M	10.5	13.5	20.75	7.25

20	M	11	14.75	24.75	10

21	M	9.5	12.25	18.75	6.5

22	M	10	13.75	22.25	8.5


C = patients controls; T = tremor group; D = dysmetria group; M = mixed group (one patient could not tolerate the test).

### Magnetic stimulation

The mean and 95% confidence intervals for the CMCTs for the four groups were: MS-C = 8.1 [7.4–8.8] ms, MS-T = 9.2 ms [8.2–10.5 ms], MS-D = 11.7 [9.7–14.9] ms and MS-M = 18.4 [16.5–20.5]. The REML analysis showed a significant effect of group with motor conduction times to increasing across the four groups from the MS controls, to MS tremor, to MS with pure dysmetria to MS with mixed tremor and dysmetria (p < 0.001). Individual data is unavailable to due to unforeseen data loss in the post-processing phase.

### Stretch Reflexes

**Figure 2:**
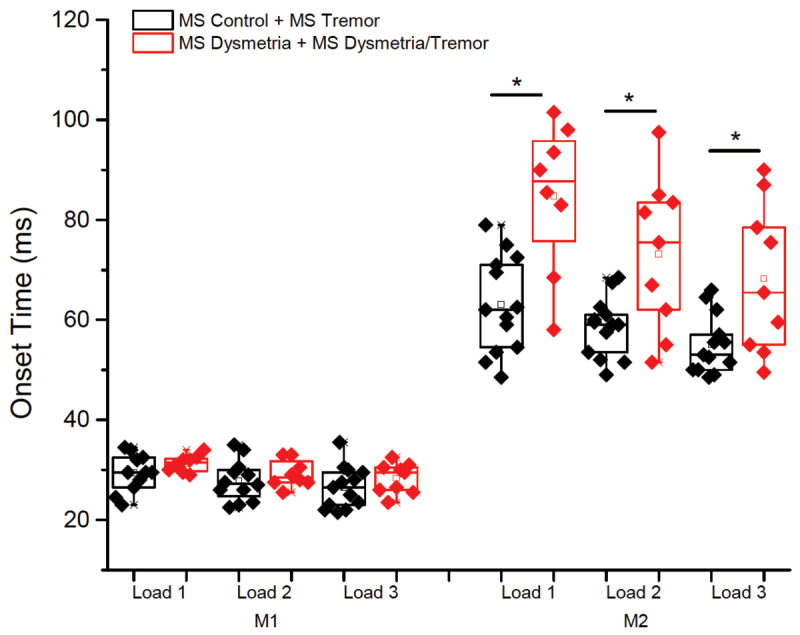
Onset latencies of the short-latency (M1) and long-latency (M2) components of the stretch reflex. Data are shown for each preload (load 1, 2 and 3) condition. The MS groups have been combined into those with dysmetria (red) and without dysmetria (black). * = p < 0.008.

There were no significant main effects of group (p > 0.0.656) or torque pre-load condition and no significant interaction (p > 0.653). Onset latencies were significantly shorted for the higher magnitude torques. M1 onset latency was not significantly different between the groups with and without dysmetria (p = 0.253) (***Figure 2***). In contrast, for M2 onset latency there was a main effect of group (F = 5.720, p = 0.007), torque pre-load (F = 29.33, p < 0.001) but no significant interaction effect (p = 0.052). The M2 onset was delayed an average of 16.5 ms (28%) in the group with dysmetria compared to the group without dysmetria. M2 onset latencies decreased with increasing torque pre-load (load 1 = 73.9 ± 2.6 ms; load 2 = 65.7 ± 2.2; load 3 = 61.2 ± 2.5 ms). There were no significant differences between groups in the magnitude of the M2 responses (normalized to baseline EMG) (p > 0.233).

## Discussion

The main finding from these experiments was the observation that conduction times (central sensory conduction, MEPs and long-latency stretch reflexes) were significantly delayed in MS patients with dysmetria, irrespective of the presence of tremor. The following discussion describes the putative pathways and mechanisms contributing to tremor and dysmetria in people with MS.

Tremor is characterized by bursts of EMG activity in the affected muscles when the normal continuous pattern of muscle activation is replaced by relatively synchronous bursting [21]. The pathophysiologic mechanisms underlying tremor may involve central or peripheral mechanisms or an interaction between the two. MS tremor is an action tremor which is thought to result from lesions in the cerebellum and/or its connections [22]. Cerebellar tremor involves a central motor loop [23], whereby central oscillators undergo spontaneous oscillation and send out rhythmic motor commands [21]. Accordingly, the generators of cerebellar tremor might be largely independent from conduction delays in peripheral, dorsal column medial lemniscal thalamocortical, and/or corticospinal pathways.

Consistent with this idea, peripheral conduction times, based on both SEPs (Erbs point and cervical evoked potentials) and short-latency reflexes, were not significantly different between MS groups, demonstrating that lesions in central pathways contribute to the pathogenesis of tremor. Central sensory conduction times of greater than 7.1 ms [19] were seen in subjects in all four subgroups (4/8 subjects in MS-C, 2/6 in MS-T, 3/4 in MS-D and 4/5 in MS-M), but there was effectively no difference between subjects with no overt movement disorder (MS-C) and those with pure tremor (MS-T). Four of six MS-T subjects had cortical SEPs and central sensory conduction times well within the normative range. Similarly, onset timing of MEPs and long-latency stretch reflexes were comparable between the MS-C and MS-T groups. Lesions of the cerebellum and/or cerebellar output pathways can result in abnormalities in the magnitude and timing of agonist-antagonist muscle activation and the generation of terminal oscillations in response to imposed perturbations, but are not associated with prolongation of M2 response latencies in the stretched muscle [17]. Thus, taken together, these data support the idea that tremor in MS is principally generated by central oscillation mechanisms likely involving cerebello-thalamocortical pathways.

In contrast, significant delays in central sensory conduction, MEPs and long-latency stretch reflex onset were seen in the subjects with dysmetria. Central sensory conduction times were an average of 29% longer in the patients with dysmetria compared with those without dysmetria. In the group with pure dysmetria, central motor conduction times were 44 and 27% longer when compared with the MS-C and MS-T groups respectively. Central motor conduction delays were particularly pronounced in patients with both tremor and dysmetria (MS-M group) with conduction times that were an average of two-fold greater than the MS-C and MC-T group and 60% longer than the pure dysmetria group. Similarly, the onset of the long latency component (M2) of the stretch reflex was an average of 28% longer in the patients with dysmetria. M2 timing represents both the ascending sensory and descending components of a transcortical pathway [24], thus, the increased latency in the patients with dysmetria likely reflects combined lesions affecting both central sensory and motor conduction but could involve differences in sensorimotor cortical processing times. Similar to the central motor conduction data, the delay in M2 onset was more marked in the group with both tremor and dysmetria (an average of 16 ms longer than the pure dysmetria group across pre-load conditions). Taken together, these data show that the presence of dysmetria in MS is associated with delays in both central sensory and motor conduction, and that slowing of central motor conduction is exacerbated in patients with both dysmetria with tremor. The co-expression of dysmetria and tremor, with worsening of central conduction delays, suggests there is more widespread CNS demyelination in these MS patients affecting conveyance of sensory information to the cortex and cerebellum, the production of motor output and the control of central cerebellothalamcortical pathways. Two regions that demonstrate oscillatory behavior within the central motor pathway are the olive and thalamic relay nuclei [21]. They are therefore possible candidates for the origin of tremors. In MS, more than one type of tremor is described; it is difficult to know if they stem from the same central oscillation [15]. Slow frequency tremors which are usually associated with dysmetria clinically, may have a different origin from those pure tremors which have a faster frequency and a more distal component. It has been postulated that tremor with different frequencies may originate from the same basic brain deficit but are modified by various inter-neuronal interactions in the brain [21]. The cells of the inferior olive can interact to produce an 8–10 Hz oscillation, which is thought to be responsible in certain models to produce a fast frequency tremor. This has been a putative but uncertain mechanism underlying essential tremor [21]. This model of tremor may explain the aetiology of the fine symmetrical distal tremor in MS, which resembles essential tremor but with a lower frequency. Slowed conduction in the motor pathways, which was demonstrated by delayed CMCT in the pure tremor group, as compared to controls, could modify the oscillation to produce a lower frequency but otherwise similar tremor to that seen in essential tremor. Alternatively slow CMCT may just reflect a more widespread demyelination. This study did not look at correlations of the findings with measures of disease severity such as the EDDS and hence conclusions are limited.

The tremorogenic role of the thalamus, which is part of a central oscillatory system, is probably more relevant to lower frequency tremors. Hyperpolarized thalamic neurons relay nuclei fire at 4–6 Hz. These relay nuclei are reciprocally connected to the reticular thalamic nuclei. When the latter nuclei receive input from the relay nuclei, they exert a powerful inhibition of them. Stimulating the cerebellar relay nuclei within the thalamus could lead to inhibition of ongoing EMG activity [25]. This inhibitory action of the thalamus may well be implicated in the generation of severe postural tremor seen in MS with relatively low frequency (3–4 Hz). The success of damping tremor by thalamotomy provides some support for this view [C], as VOP thalamotomy interrupts the basal ganglia input to the thalamus restoring the balance between competing basal ganglia-thalamic-cortical and cerebellar-brainstem-thalamic loops.

Dysmetria is likely to result from abnormalities in the timing and/or magnitude of agonist and antagonist muscle activation [5,26]. Abnormalities in the timing of sensory afferent feedback, corticomotor conduction, sensorimotor cortical processing or alterations in the output of cerebellar pathways can contribute to disruption of muscle activation. Lesions of the deep cerebellar nuclei and related output pathways can lead to abnormalities in muscle activation resulting in terminal oscillations [6,7,27]. These oscillations are due to delayed activation of the antagonist and/or second agonist burst which typically act to dampen endpoint oscillations during reaching movements. In patients with ET, the second agonist burst is significantly delayed during ballistic movements to a target, resulting in terminal oscillations and endpoint tremor, but these patients do not have dysmetria. SEP latencies are typically normal in people with ET [28]. Similarly, cerebellar lesions can lead to abnormalities in agonist-antagonist muscle timing and magnitude in response to imposed joint displacements, but the onset of the M2 component of the stretch reflex is typically unaffected [17]. In the present study, M2 onset latencies were increased in patients with dysmetria and not those with pure tremor. Furthermore, the magnitude of both M1 and M2 components of the long latency response were not significantly different between groups suggesting that scaling of muscle responses was preserved. In contrast, central sensory and motor conduction times are significantly delayed in some forms of ataxia (e.g., Friedrich’s ataxia, spinocerebellar ataxia type 1). These patients also show abnormalities in the timing of long-latency stretch reflexes [29,30]. Our findings are consistent with the idea that increased central delays are more likely to result in the expression of dysmetria than tremor in people with MS, although this central conduction delay could also be an indication of severe demyelination which may result in another abnormal circuitry directly responsible for the emergence of dysmetria. Accordingly, tremor is mediated by more central mechanisms with less of a peripheral component whereas dysmetria may be more influenced by stretch reflex corrections. If stretch reflex timing is delayed, then terminal oscillations and past pointing can occur.

The presence of upper limb tremor (without dysmetria) in MS was associated with a minimal delay (mean 1.1 ms) in the CMCT compared to MS patients with no upper limb movement disorder. It is possible that this slight mistiming induces faulty feedback to a central processor, probably in the cerebellum, which is misled into inducing tremor in some patients.

There are some limitations to this study. Firstly, the number of patients in this study is relatively small and hence this study may be considered as a pilot study which could potentially inform a larger study with prior power calculations, this is particularly important as the small number of subjects here made it difficult to know if an outlier value may be a true variability. Secondly, the patients who had tremor were not classified to those with high frequency, low amplitude ET like tremor from those with low frequency, large amplitude tremors which are usually associated with dysmetria. Furthermore, physiological data of the tremors may have also helped. Thirdly, no correlation with disease severity, such as the EDSS was carried out here. However, despite these limitations the authors believe that the study adds to the understanding of the pathophysiological processes underlying tremor and dysmetria in the context of multiple sclerosis.

In conclusion, dysmetria in MS is associated with delays in central conduction of sensory or motor pathways, or both, likely leading to disruption of muscle activation timing and terminal oscillations that contribute to dysmetria. The presence of dysmetria in MS seems to be associated with increased conduction velocities in central sensory and/or motor pathways likely reflects greater demyelination of these axons compared to those with no movement disorder or pure tremor.
